# Role of Neuraminidase in Influenza A(H7N9) Virus Receptor Binding

**DOI:** 10.1128/JVI.02293-16

**Published:** 2017-05-12

**Authors:** Donald J. Benton, Stephen A. Wharton, Stephen R. Martin, John W. McCauley

**Affiliations:** a Worldwide Influenza Centre, Francis Crick Institute, London, United Kingdom; b Structural Biology Science Technology Platform, Francis Crick Institute, London, United Kingdom; Hudson Institute of Medical Research

**Keywords:** hemagglutinin, influenza A virus, neuraminidase, receptor analogues, receptor binding, biophysics, enzyme kinetics

## Abstract

Influenza A(H7N9) viruses have caused a large number of zoonotic infections since their emergence in 2013. They remain a public health concern due to the repeated high levels of infection with these viruses and their perceived pandemic potential. A major factor that determines influenza A virus fitness and therefore transmissibility is the interaction of the surface glycoproteins hemagglutinin (HA) and neuraminidase (NA) with the cell surface receptor sialic acid. Typically, the HA is responsible for binding to the sialic acid to allow virus internalization and the NA is a sialidase responsible for cleaving sialic acid to aid virus spread and release. N9 NA has previously been shown to have receptor binding properties mediated by a sialic acid binding site, termed the hemadsorption (Hb) site, which is discrete from the enzymatically active sialidase site. This study investigated the N9 NA from a zoonotic H7N9 virus strain in order to determine its possible role in virus receptor binding. We demonstrate that this N9 NA has an active Hb site which binds to sialic acid, which enhances overall virus binding to sialic acid receptor analogues. We also show that the N9 NA can also contribute to receptor binding due to unusual kinetic characteristics of the sialidase site which specifically enhance binding to human-like α2,6-linked sialic acid receptors.

**IMPORTANCE** The interaction of influenza A virus glycoproteins with cell surface receptors is a major determinant of infectivity and therefore transmissibility. Understanding these interactions is important for understanding which factors are necessary to determine pandemic potential. Influenza A viruses generally mediate binding to cell surface sialic acid receptors via the hemagglutinin (HA) glycoprotein, with the neuraminidase (NA) glycoprotein being responsible for cleaving the receptor to allow virus release. Previous studies showed that the NA proteins of the N9 subtype can bind sialic acid via a separate binding site distinct from the sialidase active site. This study demonstrates for purified protein and virus that the NA of the zoonotic H7N9 viruses has a binding capacity via both the secondary binding site and unusual kinetic properties of the sialidase site which promote receptor binding via this site and which enhance binding to human-like receptors. This could have implications for understanding human-to-human transmission of these viruses.

## INTRODUCTION

Influenza A(H7N9) viruses first emerged as a human infection in March 2013. These viruses have been responsible for >1,250 confirmed infections, with a case fatality rate in the region of 25% (1). These infections have occurred in five separate waves, one each year since 2013. The infection of humans with these viruses is zoonotic, with very limited evidence of human-to-human transmission (2). Due to the ability of these viruses to infect humans and cause severe disease, their transmission characteristics are therefore of interest in order to assess their pandemic potential.

Major factors determining virus fitness and therefore the transmissibility of influenza A virus are the characteristics of the two surface glycoproteins, hemagglutinin (HA) and neuraminidase (NA), which control the interaction of the virus with the cell surface. These two proteins have antagonistic activities, with the HA being responsible for binding to the receptor sialic acid on the surface of cells, whereas the NA is a sialidase responsible for releasing sialic acid from glycoprotein and glycolipid sialoconjugates, to which virus may be bound, to aid virus release. A large number of previous studies have investigated the interdependence of these two activities (reviewed in reference 3).

NA from certain virus subtypes, particularly N9, have been shown to have a secondary sialic acid binding site, the hemadsorption (Hb) site, in addition to the catalytic sialidase site (4, 5). The presence of sialic acid binding via this Hb site has been shown to enhance the catalytic rate for cleavage of large multimeric substrates, such as heavily sialylated glycoproteins (5). The role of this Hb site in virus receptor binding remains unclear. Certain N2 NAs have also been found to have receptor binding properties, which occur via the sialidase site of the NA due to the substitution D151G (6). These D151G mutant NAs were found to have receptor binding properties due to their unusual enzymatic properties, with a low *K_m_* and a low *k*_cat_ indicating that the NA binds the substrate with greater strength but with lower rates of enzymatic cleavage, meaning that the NA acquires more of a receptor binding role (7).

The receptor binding properties of a zoonotic H7N9 virus have previously been characterized by biolayer interferometry (BLI) (8). This and other studies (9–15) indicate that the H7 HA has an overall preference for binding to avian-like α2,3-linked sialic acid receptors, but the viruses also show considerable binding to the human-like α2,6-linked sialic acid receptors. It is unclear, however, how these binding characteristics affect the ability of these viruses to result in zoonotic infection but not transmit between humans. This study investigates the receptor binding and enzymatic characteristics of the N9 NA from a prototype H7N9 virus with an aim to elucidate a possible role of the NA in affecting virus binding properties. We present biophysical data, obtained using previously characterized techniques (16), that show that the N9 NA can bind to sialic acid via the secondary Hb site and that the sialidase site enhances virus binding to sialic acid, particularly to human-like α2,6-linked receptors. The enhancement of binding seen with this particular NA has implications for understanding the fitness and therefore the transmissibility of these viruses.

## RESULTS AND DISCUSSION

### Binding properties of recombinant N9 protein.

**FIG 1:**
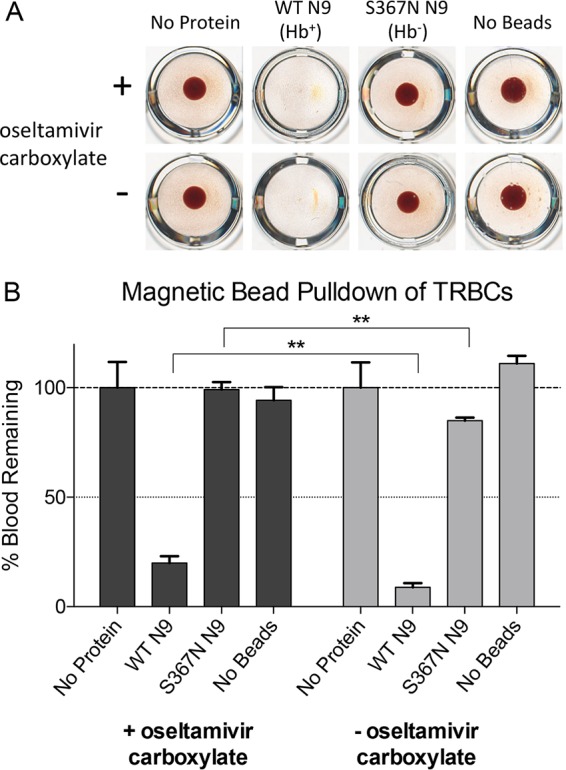
Red blood cell capture assay by WT (Hb^+^) and S367N (Hb^−^) N9 NAs. Insect cell-expressed N9 NA was attached to His tag binding magnetic beads. The beads were incubated with turkey red blood cells (TRBCs) and rapidly pelleted with a magnet. The supernatant was removed and left to settle in a microtiter plate (A), or TRBCs were lysed with SDS and the released hemoglobin was quantified by measuring the *A*_540_ (B). The results of control experiments without protein attached to beads (no protein) and without incubation of beads with TRBCs (no beads) are also shown. Absorbance measurements were normalized to those for the no-protein control and are shown as the means from three independent measurements, with error bars showing standard deviations from the mean. When present, oseltamivir carboxylate was added at a concentration of 100 μM. **, *P* < 0.01.

Experiments were carried out to determine whether the N9 NA of the H7N9 viruses infecting humans has receptor binding properties. The N9 NA of A/Anhui/1/2013 (Anhui13) was expressed in insect cells as the wild type (WT) and as a previously characterized S367N mutant, which is known to abolish sialic acid binding via the Hb site (5). These expressed proteins were attached to His tag binding magnetic beads and used to measure the ability of the protein to capture turkey red blood cells (TRBCs). NA-coated beads were incubated with TRBCs, and the beads were rapidly pelleted using a magnet. The blood remaining in the supernatant either was left to settle in a microtiter plate (Fig. 1A) or was lysed, and the relative hemoglobin concentration was determined by absorbance spectroscopy (Fig. 1B). These assays demonstrated the ability of WT NA to bind to TRBCs, thus demonstrating its sialic acid binding properties. This binding was abolished when the substitution S367N was introduced, demonstrating that the majority of the binding seen is mediated through the Hb site. Similar levels of binding by the N9 NA were seen in the presence and absence of the sialidase site inhibitor oseltamivir carboxylate, ruling out binding of this inhibitor to the Hb site. There was, however, enhanced capture of TRBCs, indicated by a reduced level of residual TRBCs following capture by the NA-coated beads, when the sialidase site of the mutant NA was not inhibited for both the WT and S367N proteins (Fig. 1B), suggesting a possible contribution of this enzyme active site to receptor binding.

### Virus binding properties.

A number of different viruses which contained the NA from Anhui13 in both the wild-type form (which bound via the Hb site [Hb^+^ NA]) and the S367N mutant form (which lacked binding via the Hb site [Hb^−^ NA]) were generated by reverse genetics to examine the contribution of the Hb site to receptor binding. The viruses generated consisted of H7N9 viruses with the HA from Anhui13, H1N9 viruses with the HA from A/Puerto Rico/8/34 (PR8), and H3N9 viruses with the HA derived from the recent cell culture-propagated cultivar of A/Victoria/361/2012 (Vic361) H3N2 virus (17).

**FIG 2:**
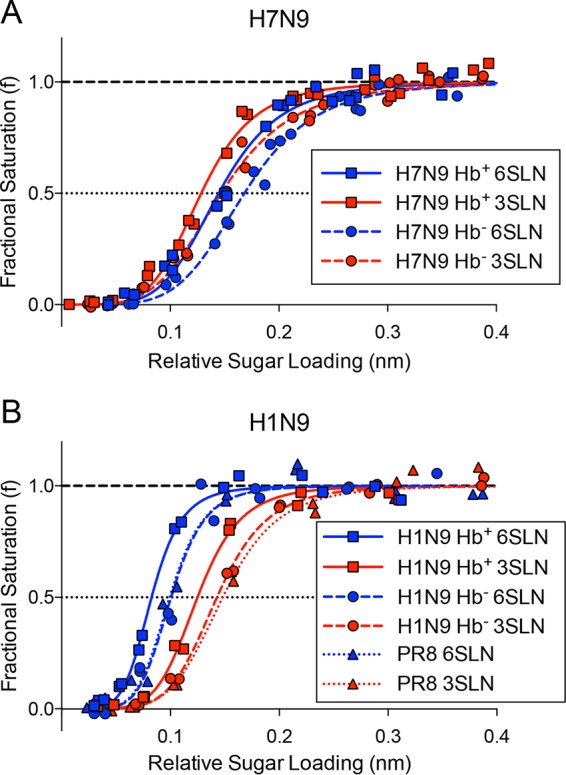
Biolayer interferometry curves of influenza A virus (100 pM) binding to the receptor analogues α2,6-sialyl-*N*-acetyllactosamine (6SLN) and α2,3-sialyl-*N*-acetyllactosamine (3SLN). The binding of H7N9 (A) and H1N9 (B) viruses to both wild-type (Hb^+^) and S367N mutant (Hb^−^) N9 NAs was measured. The plotted data are the fractional saturation of virus binding as a function of sugar loading.

The equilibrium receptor binding characteristics of these viruses were determined by biolayer interferometry (BLI), measuring virus binding as a function of relative sugar loading (RSL) in the presence of NA inhibitors, as previously described (18). Figure 2 shows the results for H7N9 and H1N9 virus binding to sialoglycopolymers bearing the human-like α2,6-sialyl-*N*-acetyllactosamine (6SLN) and avian-like α2,3-sialyl-*N*-acetyllactosamine (3SLN) receptor analogues. The observed binding of the H7N9 virus was similar to previously reported BLI data for binding of the wild-type virus (8), with an overall preference for binding to the avian-like receptor 3SLN and marginally weaker binding to the human receptor analogue 6SLN. The H7N9 and H1N9 viruses showed an enhancement of binding to both 6SLN and 3SLN when the Hb^+^ NA was present, giving an estimated decrease in the relative *K_d_*_(virus)_ (dissociation constant for the virus) of ∼2-fold. The H1N9 virus with Hb^−^ NA (H1N9 Hb^−^ virus) showed levels of binding similar to those of the full 8-segment PR8 virus (Fig. 2B), indicating that the mutation (S367N) reduces binding to a level which is very close to that of the control virus, PR8, without Hb binding in the NA. Both H7N9 and H1N9 viruses showed similar increases in binding to both the α2,6- and α2,3-linked receptors when the Hb^+^ NA was present. This indicates that there is no receptor linkage preference associated with the presence of the Hb^+^ NA.

**FIG 3:**
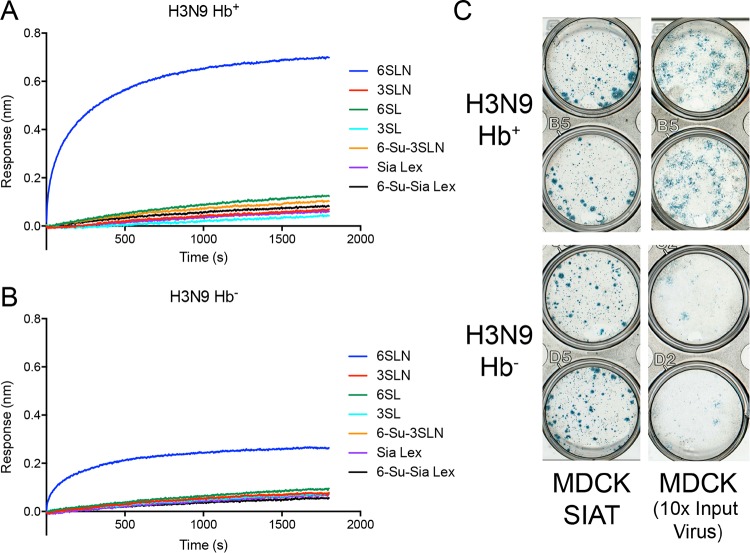
(A and B) Binding of H3N9 viruses to Hb^+^ (A) and Hb^−^ (B) NAs determined by biolayer interferometry. Binding to a range of different sugars was measured: α2,6- and α2,3-sialyl-*N*-acetyllactosamine (6SLN and 3SLN, respectively), α2,6- and α2,3-sialyllactose (6SL and 3SL, respectively), 3SLN 6′ sulfated on GlcNAc (6-Su-3SLN), sialyl-Lewis X (Sia Lex), and sialyl Lewis X 6′ sulfated on GlcNAc (6-Su-Sia Lex). The data shown are experimental response traces. (C) Plaque assay of H3N9 viruses with Hb^+^ and Hb^−^ NAs. The results of assays carried out using both MDCK and MDCK-SIAT cells are shown in duplicate. The plaques shown for MDCK cells used 10 times the concentration of each input virus compared to that used for MDCK-SIAT cells.

H3N9 viruses with the HA from Vic361 were constructed in order to assess whether the weak binding to human-like receptor analogues of these recent H3N2 viruses (17, 18) could be enhanced by the addition of an Hb^+^ NA. Binding of these viruses is weak, and only binding to 6SLN is observed; consequently, measurements had to be made at 50 times the normal virus concentration (5 nM). At this concentration, the presence of the Hb^+^ NA gave an increase in binding to 6SLN (Fig. 3A and B), but no binding to any other receptors tested was observed. This increase in binding amplitude to a saturation level of ∼0.75 nm is, however, small compared to the binding amplitude in similar experiments for H1N9 and H7N9 viruses, which give signals of >4 nm at saturation. This apparent preference for 6SLN is likely not due to a specificity of the N9 Hb site for 6SLN, but rather, the binding via the Hb site complements the weak binding of the HA, which is to 6SLN. It is notable that viruses bearing the HA from recent H3N2 viruses normally require MDCK-SIAT cells for efficient replication (6, 19); these cells have enhanced expression of α2,6-linked cell surface receptors (20). The propagation characteristics of Hb^+^ and Hb^−^ H3N9 viruses were similar in MDCK-SIAT cells (Fig. 3C); however, the Hb^+^ virus had enhanced replication in MDCK cells, indicating that the Hb site can enhance binding to the insufficient receptors present on MDCK cells.

### HA/NA balance characteristics.

**FIG 4:**
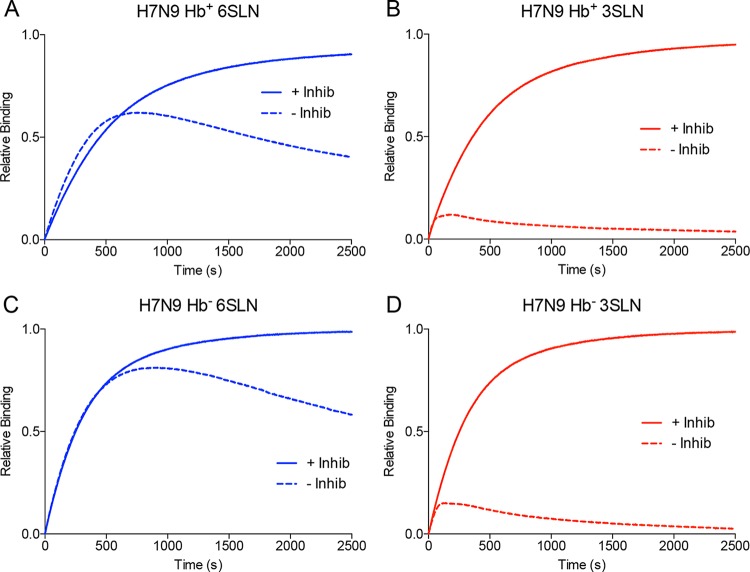
Biolayer interferometry curves of binding of H7N9 viruses with wild-type (Hb^+^) and S367N (Hb^−^) NA to the receptor analogues α2,6-sialyl-*N*-acetyllactosamine (6SLN) and α2,3-sialyl-*N*-acetyllactosamine (3SLN). Measurements were made in the presence (solid lines) and absence (dashed lines) of NA inhibitors (Inhib).

The balance between the activities of the HA and NA was measured using a approach developed previously (16). In these experiments, the binding of viruses was measured both in the presence and in the absence of NA inhibitors. Figure 4 shows the binding of H7N9 viruses with Hb^+^ and Hb^−^ NA binding to the human-like 6SLN and the avian-like 3SLN receptors. The binding of the Hb^+^ and Hb^−^ H7N9 viruses to 3SLN showed behavior (Fig. 4B and D) similar to that measured previously with the H3N2 virus X-31 (16), with the uninhibited virus showing very little binding, presumably due to efficient cleavage of the α2,3-linked receptor by the NA. Binding to the human-like receptor 6SLN showed different characteristics. In the absence of NA inhibitors, the H7N9 Hb^+^ virus showed an initial enhancement of binding at short times (<500 s) which was not seen with the Hb^−^ virus (Fig. 4A and C). This enhancement of binding, when taken at a single time point of 250 s, provided a 23% higher binding signal in the absence of NA inhibitors. This binding enhancement was seen only when the Hb site was present and when the sialidase site was uninhibited. Although it is likely that both the sialidase site and the Hb site contribute to this enhancement of binding, it is not possible to assess the relative importance of the two sites with the data obtained. The binding of the H7N9 Hb^+^ virus to 6SLN in the absence of NA inhibitors showed a lower fractional saturation maximum (∼0.6) than did that of the Hb^−^ virus (∼0.8) (Fig. 4A and C), suggesting that the presence of the Hb site could enhance the ability of the NA to cleave multimeric substrates, as previously reported (5).

**FIG 5:**
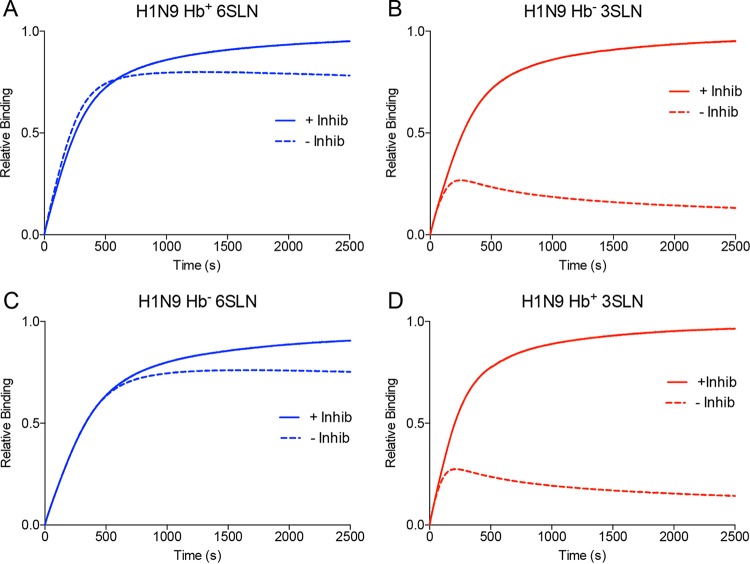
Biolayer interferometry curves of H1N9 virus (100 pM) binding to the receptor analogues α2,6-sialyl-*N*-acetyllactosamine (6SLN) and α2,3-sialyl-*N*-acetyllactosamine (3SLN). Measurements were made in the presence (solid lines) and absence (dashed lines) of NA inhibitors. The binding of viruses to wild-type (Hb^+^) and S367N (Hb^−^) N9 NAs was measured.

A similar enhancement of binding to 6SLN was observed when the H1N9 Hb^+^ and Hb^−^ viruses were compared (Fig. 5), although the enhancement of binding was smaller than that seen for the H7N9 viruses, likely due to the higher affinity of the PR8 HA for 6SLN (Fig. 2B), which would lead to a smaller proportional binding contribution of the NA to these viruses.

### NA kinetics.

The data presented above for virus binding indicate that the N9 NA enhances overall receptor binding in the context of a number of different viruses. This binding is mediated by both the Hb and the sialidase sites of the NA. The binding mediated through the sialidase site appears to preferentially result in binding to the α2,6-linked receptor 6SLN. It is, consequently, important to identify the kinetic parameters for different receptors to understand these unusual binding characteristics of the N9 NA.

Enzyme kinetic parameters were determined for expressed Hb^+^ and Hb^−^ Anhui13 NAs. The cleavage of the fluorogenic substrate 2′-(4-methylumbelliferyl)-α-d-*N*-acetylneuraminic acid (MUNANA) was the same for both the Hb^+^ and Hb^−^ NAs, indicating that there is no influence of the Hb site on the ability of the NA to cleave small monomeric substrates (Table 1).

**TABLE 1:** Steady-state kinetic properties of N9 NAs

Substrate	NA protein	Mean ± SE *K_m_* (μM)	Mean ± SE *k*_cat_ (s^−1^)	*k*_cat_/*K_m_* (μM^−1^ s^−1^)
MUNANA	Hb^+^	137.9 ± 12.8	90.1 ± 4.7	0.654
MUNANA	Hb^−^	134.2 ± 12.9	88.6 ± 4.7	0.660
6SLN	Hb^+^	4,179 ± 615	2.2 ± 0.2	0.000537
3SLN	Hb^+^	810 ± 100	159.5 ± 8.5	0.197
6SLN	X-31^*a*^	8,070 ± 615	18.5 ± 0.9	0.00229
3SLN	X-31^*a*^	562.3 ± 20	97.5 ± 1.5	0.173
Fetuin	Hb^+^	197.5 ± 37.3	71.6 ± 8.3	0.363
Fetuin	Hb^−^	346.4 ± 95.6	73.0 ± 14.5	0.211

a The values were determined in a previous study (16).

The cleavage of the monomeric avian-like receptor 3SLN is efficient, with a relatively low *K_m_* and a high *k*_cat_, giving results similar to those previously determined for the NA of X-31 (Table 1) (16). The characteristics of cleavage for the human-like receptor 6SLN are, however, unusual compared to those previously obtained for the NA of the H3N2 virus X-31. The *K_m_* was ∼2-fold lower (4,179 ± 615 μM for Anhui13, 8,070 ± 615 μM for X-31), indicating stronger substrate binding. The *k*_cat_ of the N9 NA was also ∼10-fold lower than that of the X-31 NA (2.2 ± 0.2 s^−1^ for Anhui13, 18.5 ± 0.9 s^−1^ for X-31), indicating a reduced enzymatic turnover (16). The combination of these two parameters shows that the N9 NA can bind to α2,6-linked receptors more strongly but cleaves them with a lower efficiency than does X-31, suggesting that the N9 NA has the ability to play a receptor binding role. If one assumes a reasonable value for the association constant (*k*_1_) of sialic acid for the NA sialidase site of 5 × 10^5^ M^−1^ s^−1^ (21), the substrate dissociation constant (*k*_−1_) can be calculated using the *K_m_* and *k*_cat_ values determined for the N9 NA (*k*_−1_ = *K_m_* · *k*_1_ − *k*_cat_). Using the calculated *k*_−1_ and the experimentally determined *k*_cat_, the probability that bound substrate will be cleaved or will dissociate before undergoing cleavage can be determined by calculating the ratio between *k*_cat_ and *k*_−1_. The N9 NA is ∼1,000-fold more likely to dissociate rather than cleave 6SLN, whereas the likelihood for 3SLN is ∼1.5-fold. The comparable probability values for another NA previously characterized from the H3N2 virus X-31 (16) are an ∼200-fold probability of 6SLN dissociation rather than cleavage and an ∼1.8-fold probability for 3SLN dissociation rather than cleavage. Therefore, the N9 NA has an ∼5-fold enhanced probability of 6SLN dissociation compared to the NA of X-31, and the probability value for 3SLN is similar. This enhanced probability of 6SLN dissociation rather than cleavage coupled with a relatively low *K_m_* compared to that of the X-31 NA reinforces the hypothesis that substrate binding via the sialidase site is at least partially responsible for the enhancement of the initial binding to 6SLN by H7N9 and H1N9 viruses with the WT Hb^+^ NA (Fig. 4 and 5).

Cleavage of the multimeric substrate fetuin by the Hb^+^ NA has a *K_m_* (197.5 ± 37.3 μM) lower than that measured for the Hb^−^ NA (346.4 ± 95.6 μM), indicating that the presence of the Hb site increases the affinity of the NA for multimeric substrates. However, the *k*_cat_ values for the Hb^+^ and the Hb^−^ NAs are similar, indicating that there is no difference in enzyme turnover when the substrate concentration is not limiting. The Hb^−^ NA has an overall catalytic efficiency (*k*_cat_/*K_m_*) which is ∼70% lower than that of the Hb^+^ NA, indicating increased efficiency when the Hb site is present, as has been previously reported for experiments carried out with N2 modified to have Hb binding properties (5).

### Virological significance.

It has been noted in the past that the protein motifs that confer Hb activity are present in a wide range of virus subtypes which are predominantly from avian sources (4, 22). It is yet unclear whether the specific kinetic properties of the N9 NA relating to the sialidase site, which favor the release rather than the cleavage of the receptor in an α2,6-linked-specific manner, are also an inherent characteristic of avian influenza viruses in general and whether these are associated with an avian host tropism.

There are potentially interesting consequences for the H7N9 viruses having Hb properties associated with the NA. The increase in overall sialic acid binding of these viruses and the enhanced binding to α2,6-linked sialic acid mediated by the sialidase site are likely advantages for the initial receptor binding to initiate infection, as the enhanced binding would increase the residence time of viruses at the surface of the cells of the human upper respiratory tract, which are rich in α2,6-linked receptors, thus likely increasing the probability of infection. This is the first reported example of the virus receptor binding specificity being determined, in part, by the NA. The N9 NA is, however, poor at cleaving α2,6-linked receptors. There are, consequently, likely to be problems relating to the inefficient release of virus from human cells after replication. This inefficient release could be a factor limiting the efficiency of aerosol transmission in ferrets (11, 23–25) and human-to-human transmission of the virus (2).

## MATERIALS AND METHODS

### Viruses.

All viruses used were constructed by reverse genetics based on a previously published system, with cDNAs for each gene segment being cloned into the pHW2000 vector (26). All viruses were generated in a 6 + 2 reassortment using the HA and NA genes from the desired virus and the remaining 6 gene segments from A/Puerto Rico/8/34(H1N1) (PR8). The H7N9 viruses were constructed using the HA and NA genes of A/Anhui/1/2013(H7N9) (Anhui13), the H1N9 viruses had the HA from PR8 and the NA from Anhui13, and the H3N9 viruses had the HA from a cell culture-propagated cultivar of A/Victoria/361/2012(H3N2) (Vic361) and the NA from Anhui13. To make viruses lacking binding via the Hb site (Hb^−^ viruses), the NA substitution S367N was introduced into Anhui13 NA by QuikChange mutagenesis (Agilent). All viruses were rescued by the cotransfection of the 8 desired plasmids in 293T cells. H7N9 viruses were propagated in MDCK cells and H3N9 viruses were propagated in MDCK-SIAT cells in serum-free medium in the presence of 2 μg/ml tosylsulfonyl phenylalanyl chloromethyl ketone (TPCK)-trypsin (Sigma). H1N9 viruses were propagated in 11- to 12-day-old embryonated hens' eggs. Cell culture-propagated viruses were concentrated by pelleting, and egg-propagated viruses were purified through sucrose gradients, as described previously (16). The concentration of purified/concentrated virus was determined by a solid-phase NP enzyme-linked immunosorbent assay as previously described (18). The generation, propagation, and subsequent experiments involving H7N9 and H3N9 viruses were carried out under an appropriate high biological containment level. All viruses were sequenced to ensure that no changes in the HA or NA sequence had occurred upon propagation. Plaque assays were performed with either confluent MDCK or MDCK-SIAT cells using 1.2% (wt/vol) Avicel microcrystalline cellulose and 2 μg/ml TPCK-trypsin in 96-well plates.

### Protein expression and purification.

An expression construct containing genes encoding the Anhui13 NA ectodomain (residues 75 to 465) with an N-terminal purification tag was synthesized (GeneArt). The purification tag consisted of a hexa-His tag, a human vasodilator-stimulated phosphoprotein tetramerization domain (27, 28), and a tobacco etch virus protease cleavage site. The synthesized construct was cloned into the pFB-LIC-Bse vector with an In-Fusion cloning kit (Clontech). The substitution S367N was introduced by QuikChange mutagenesis (Agilent). Recombinant baculovirus was generated using a Bac-to-Bac system according to the manufacturer's instructions (Life Technologies). Following virus amplification, large-scale protein expression was carried out with 2.5 liters of Sf9 cells. Cells were removed by centrifugation at 72 h after infection, and the protein in the supernatant was concentrated and loaded onto a HisTALON column (Clontech). Fractions containing NA were pooled and dialyzed against 25 mM Tris-HCl, pH 8.0, 150 mM NaCl. The NA was further purified by gel filtration using a Superdex 200-pg 16/60 column (GE) in 25 mM Tris-HCl, pH 8.0, 150 mM NaCl. The gel-filtered protein was concentrated and stored in 25 mM Tris-HCl, pH 8.0, 150 mM NaCl, 4 mM CaCl_2_, 0.01% NaN_3_.

### Red blood cell capture assay.

Expressed N9 proteins were attached to His tag isolation and pulldown Dynabeads (Life Technologies). One hundred microliters of extensively washed beads was added to 50 μl of 10 μM N9 NA (monomeric concentration), the mixture was incubated for 30 min, and then the beads were washed to remove unbound NA. Three microliters of the beads was added to 100 μl of 0.5% (vol/vol) turkey red blood cells (TRBCs), and the mixture was incubated at room temperature for 15 min. Samples were thoroughly mixed, and the beads were rapidly pelleted using a magnet. The supernatant was removed and allowed to settle in a microtiter plate for visual quantification, or TRBCs were lysed with SDS (final concentration, 0.5% [wt/vol]), and the released hemoglobin levels were assayed by measuring the absorbance at 540 nm.

### Virus binding studies.

Virus binding was measured as previously described (16). Briefly, binding was measured by biolayer interferometry (BLI) using an Octet Red system (Pall ForteBio Corp., Menlo Park, CA, USA). Streptavidin-coated sensors (Pall ForteBio Corp.) were loaded with biotinylated sialoglycopolymers, which consisted of 20 mol% sugar, either α2,6-sialyl-*N*-acetyllactosamine (6SLN) or α2,3-sialyl-*N*-acetyllactosamine (3SLN), attached to a polyacrylamide backbone with 5 mol% biotin (Lectinity Holdings, Moscow, Russia). Sugars were loaded at a range of concentrations for equilibrium binding assays. HA and NA balance measurements (16) were made using sensors saturated with receptor analogues (loading ≈ 0.6 nm). All experiments were carried out in 10 mM HEPES, pH 7.4, 150 mM NaCl, 0.005% (vol/vol) Tween 20, 4 mM CaCl_2_. When present, the NA inhibitors oseltamivir carboxylate (Roche Products Ltd., Welwyn Garden City, UK) and zanamivir (GlaxoSmithKline, Stevenage, UK) were added at concentrations of 100 μM to inhibit NA sialidase activity. Unless stated otherwise, the virus concentration was 100 pM in all binding assays. For equilibrium binding, the total amplitude of virus binding was measured and plotted as a function of sugar loading.

### NA kinetics.

Kinetic parameters for cleavage of 2′-(4-methylumbelliferyl)-α-d-*N*-acetylneuraminic acid (MUNANA), 6SLN, and 3SLN were determined as previously described (16). Cleavage of fetuin (Sigma) was measured by using a system containing *N*-acetylneuraminic acid aldolase (NANA aldolase) and lactate dehydrogenase (LDH) as a reporter of released sialic acid. The 100-μl reaction mixtures contained 0.5 U NANA aldolase (Sigma), 5 U LDH from bovine heart (Sigma), 200 μM NADH (Sigma), and fetuin from bovine serum (Sigma) at concentrations ranging from 20 to 200 μM. Measurements were made in 10 mM HEPES, pH 7.4, 150 mM NaCl, 0.005% (vol/vol) Tween 20, 4 mM CaCl_2_. Initial reaction rates were determined by measuring the reduction in absorbance at 340 nm in a Jasco V-550 spectrophotometer using an ultramicrovolume 10-mm-path cuvette at 37°C. This change in absorbance was converted to the change in the molar concentration of NADH using its extinction coefficient (ε = 6,220 M^−1^ cm^−1^).
